# Animal Harms and Food Production: Informing Ethical Choices

**DOI:** 10.3390/ani11051225

**Published:** 2021-04-23

**Authors:** Jordan O. Hampton, Timothy H. Hyndman, Benjamin L. Allen, Bob Fischer

**Affiliations:** 1 Faculty of Veterinary and Agricultural Sciences, University of Melbourne, Parkville, VIC 3052, Australia; 2 School of Veterinary Medicine, Murdoch University, Murdoch, WA 6150, Australia; t.hyndman@murdoch.edu.au; 3 Harry Butler Institute, Murdoch University, Murdoch, WA 6150, Australia; 4 Institute for Life Sciences and the Environment, University of Southern Queensland, Toowoomba, QLD 4350, Australia; benjamin.allen@usq.edu.au; 5 Centre for African Conservation Ecology, Nelson Mandela University, Port Elizabeth 6034, South Africa; 6 Department of Philosophy, Texas State University, San Marcos, TX 78666, USA; fischer@txstate.edu

**Keywords:** agriculture, animal welfare, ethics, harms, harvesting, hunting, ranking, wildlife

## Abstract

**Simple Summary:**

Consideration of animal welfare in food choices has become an influential contemporary theme. Traditional animal welfare views about food have been largely restricted to direct and intentional harms to livestock in intensive animal agriculture settings. However, many harms to animals arising from diverse food production practices in the world are exerted indirectly and unintentionally and often affect wildlife. Here we apply a qualitative analysis of food production by considering the breadth of harms caused by different food production systems to wild as well as domestic animals. Production systems are identified that produce relatively few and relatively many harms. The ethical implications of these findings are discussed for consumers concerned with the broad animal welfare impacts of their food choices.

**Abstract:**

Ethical food choices have become an important societal theme in post-industrial countries. Many consumers are particularly interested in the animal welfare implications of the various foods they may choose to consume. However, concepts in animal welfare are rapidly evolving towards consideration of all animals (including wildlife) in contemporary approaches such as “One Welfare”. This approach requires recognition that negative impacts (harms) may be intentional and obvious (e.g., slaughter of livestock) but also include the under-appreciated indirect or unintentional harms that often impact wildlife (e.g., land clearing). This is especially true in the Anthropocene, where impacts on non-human life are almost ubiquitous across all human activities. We applied the “harms” model of animal welfare assessment to several common food production systems and provide a framework for assessing the breadth (not intensity) of harms imposed. We considered all harms caused to wild as well as domestic animals, both direct effects and indirect effects. We described 21 forms of harm and considered how they applied to 16 forms of food production. Our analysis suggests that all food production systems harm animals to some degree and that the majority of these harms affect wildlife, not livestock. We conclude that the food production systems likely to impose the greatest overall breadth of harms to animals are intensive animal agriculture industries (e.g., dairy) that rely on a secondary food production system (e.g., cropping), while harvesting of locally available wild plants, mushrooms or seaweed is likely to impose the least harms. We present this conceptual analysis as a resource for those who want to begin considering the complex animal welfare trade-offs involved in their food choices.

## 1. Introduction

Many groups in modern societies are interested in the animal welfare consequences of food production. These groups span food producers, processors, retailers and policy makers, as well as consumers [1]. Modern consumers are particularly interested in animal welfare when it comes to the various products they may choose or choose not to purchase. This interest has become particularly evident in food consumption in recent years, in developed and developing countries [2]. However, the competing claims of marketing, fashion, industry lobbying and advocacy groups can make discerning and comparing animal welfare criteria problematic for consumers. Additionally, in the era now termed the Anthropocene, human impacts are widespread and the way in which these impacts harm animals may be obscure to consumers and producers alike. Human activities over the last 200 years have transformed the planet [3], and are forcing us to change the way we see our impacts and responsibilities. If consumers want to make more thoughtful food choices, then consumers need the ability to conceptualize and categorize those impacts.

There is also much more to food ethics than just animal welfare. Consumers are also interested in the environmental footprints of various foods [4], the related question of sustainability [5], the plight of humans involved in the production of food [6], the geopolitical consequences of different trade relationships [7], and so on. In each case, many studies have assessed the consequences of consuming different food types. For instance, there have been many analyses of different food systems comparing environmental impacts generally [8], and climate change impacts specifically [4], but we are unaware of any study to explicitly apply a similar approach to animal welfare impacts. There is little published information relating to the animal welfare impacts of food production systems that are not livestock-based, and less still to the downstream effects on various systems.

This knowledge gap may be related to the history of animal welfare science as a discipline, which is focused on the way direct and intentional actions of moral agents (humans) impact individual animals, rather than the unintentional or indirect processes that determine consequences for the largest numbers of animals [9]. This trend is displayed by the disproportionate focus of animal welfare studies on agricultural animals (i.e., livestock) [10] compared to the paucity of studies examining wildlife [11]. There is growing awareness that animal welfare should be considered in all of our relationships with animals, not only for direct impacts, but also for indirect impacts [3]. Here we attempt to go some distance toward addressing this knowledge gap by applying tenets of the “One Welfare” paradigm [12] to assess animal welfare impacts from a holistic perspective by considering all processes that may harm animals in agroecosystems. Agroecosystems are natural ecosystems that have been modified to enhance food (and fiber) production [13], and the concept emphasizes that there are many more animals and plants in these systems than those few domesticated species use to produce products of societal value. Our aim, then, was to use this paradigm to provide a summary of the ways that food production may create negative impacts for all animals.

We recognize that consumers need more than just a list of animal impacts associated with industrial activities to make ethical choices. Enumerating impacts is one task; assessing their scope and intensity is another. Assessment of harm intensity is beyond the scope of this paper, and our intent is not to show that consumers ought to eat in one way or another, even if they are exclusively concerned with animal welfare. Rather, our intent is to provide a resource for those who want to begin considering the very complex animal welfare trade-offs involved in their food choices. This is because, depending on a consumer’s philosophical persuasions, they may weigh some welfare impacts much more heavily than others, and we cannot explore all this variation here. Some consumers, for instance, may distinguish between intended and merely foreseen impacts, which would lead them to be more concerned about harms to hunted animals (which are generally intended) than harms to wild animals around agricultural lands (which are generally unintended but foreseen). Consumers may also assign different weights to animals based on their species preferences; some may weigh impacts on wild animals more heavily, which may make them particularly concerned about the wildlife trade; others may think that animals’ cognitive complexity is especially important, elevating the moral significance of impacts on mammals over most invertebrates. These and other disagreements will generate very different practical recommendations.

## 2. Animal Harms

Much animal welfare focus has traditionally been on human (anthropogenic) activities that directly and intentionally harm animals (e.g., raising livestock), but there has been much less awareness of activities that indirectly and/or unintentionally cause undesirable animal welfare outcomes [9]. These unintended effects of human activities kill and injure far more animals than many of the intentional activities that traditionally raise moral concern about animal welfare [9]. As a departure from the traditional focus of animal welfare science on intentional impacts, Fraser and MacRae [14] proposed the “harms” approach to include consideration of anthropogenic processes that harm large numbers of animals but may not be perpetrated deliberately or widely known. This approach to animal welfare does not attempt to rank impacts from “best” to “worst” but instead allows consideration of all processes that harm animals, whether they are intentional, direct or otherwise [14].

The harms model has been applied in several wildlife contexts [15], including management of hyperabundant species [16] and predation of livestock [13]. More broadly, the focus on unintentional and indirect impacts has been much better developed in the environmental sciences than in animal welfare science. For example, many consumers are aware of marketing campaigns attempting to dissuade consumers from purchasing palm oil products [17]. This advocacy does not claim that palm oil production as a form of horticulture involves any intentional harm to animals, but that the land clearing required for its expansion indirectly threatens the survival of many charismatic wildlife species, such as orangutans (*Pongo* spp.) [18]. We note that this issue is complex, and current alternatives to palm oil may well yield worse environmental outcomes [19], but the key point is that there is a public discussion around the nature and magnitude of unintentional impacts of oil production.

### 2.1. Types of Animal Harms

Fraser and MacRae [14] proposed that people affect animals through four broad types of activity:

- Type 1 harms: keeping of domestic or captive animals for companionship, livestock, entertainment, laboratory use, racing, security, etc.;
- Type 2 harms: causing deliberate harm to animals through activities such as slaughter, pest control, fishing, hunting, and toxicology testing;
- Type 3 harms: causing direct but unintended harm to animals through land clearing, window-strike, vehicle collisions, etc.; and
- Type 4 harms: negatively affecting the welfare of animals indirectly by disturbing ecological systems through processes like climate change, pollution, introducing invasive species, etc.

Animal welfare science has traditionally focused on Type 1 and 2 activities [11] but holistic approaches require further consideration of Type 3 and 4 activities to account for all processes that may affect the welfare of all animals [20]. By definition, Type 1 activities only affect domesticated animals, while Type 2 harms, although affecting both wild and domestic animals, are most commonly examined in relation to domesticity. Type 3 and 4 harms most often affect wild animals. With increasing focus on wild animal welfare, there is growing awareness of the importance of indirect harms, sometimes referred to as “invisible” harms [21].

### 2.2. Examples of Indirect and Unintentional Harms

A notable example of the importance of unintended harms can be seen in analyses of wild bird mortalities. Recent research in the USA (based on imperfect extrapolation) has suggested that the processes responsible for the most and second-most wild birds being killed or injured are predation from pet cats (an indirect or Type 4 harm) and collisions with windows (a direct or Type 3 harm), respectively. Deliberate harms such as hunting or pest control are responsible for far less cumulative animal welfare impacts to wild birds [22]. Contemporary examples of indirect harms that culminate in wild bird mortalities include the impact of wind turbines (“wind farms”), communication towers and solar arrays [22]. Beyond harms that cause mortalities, there have also been vast improvements in understanding how wild animals are affected by non-lethal interactions with anthropogenic stressors like artificial light and sound (e.g., non-lethal stress in peri-urban wildlife [23]). These non-lethal effects also constitute harms.

To our knowledge, the harms model has not previously been explicitly applied to food production systems. Although it has been used to assess specific cases (e.g., predation management in extensive beef production systems [13]) or to generally assess processes that may impact the welfare of wildlife [15]. An approach centered on minimizing harms has also been proposed for managing wildlife health [24], with close links to animal welfare. Here we apply the harms model to the food production systems that most commonly provide commercially available human food products in developed countries. We describe the breadth of harms in Section 3, provide summaries of these harms for each food production system in Section 4, compare and rank these harms in Section 5, assist with consumer decision making in Section 6, and provide recommendations for future work in Section 7.

We take examples of harms from published studies from around the world but focus on Australia, a continent possessing a wide diversity of food-producing industries [25], unique contemporary ecological challenges [26,27], and well-described anthropogenic impacts on animals [21].

## 3. Harms Relevant to Food Production

### 3.1. Type 1 Harms

All husbandry practices associated with the breeding, keeping, transport and slaughter of livestock constitute Type 1 harms. These impacts may range from those considered relatively mild (e.g., fear of humans in farmed animals [28]) to those that are often considered to be severe (e.g., force-feeding of geese for foie gras [29]). This type of harm does not apply to production systems that raise plants or fungi or those that harvest wild animals without the use of domestic animals. Keeping of laboratory animals is also a Type 1 harm, but where this use of laboratory animals relates to food production (food safety or toxicology testing), we referred to this as a Type 2 harm [14] for simplicity.

#### 3.1.1. On-Farm Husbandry

All husbandry procedures imposed on livestock arguably create animal welfare impacts. Well-described procedures include confinement (e.g., pigs in sow stalls [30]), behavioral deprivation (e.g., absence of opportunities to forage in laying hens [31]), reproductive manipulation such as adult–young (e.g., cow–calf) separation [32], painful procedures such as mulesing in sheep [33] and de-horning in cattle [34]. Numerous other routine husbandry procedures harm domestic animals and are well-described in livestock science. On-farm husbandry also applies to fish kept in freshwater (aquaculture) or marine (mariculture) contexts and the suite of welfare impacts they may experience in these conditions [35]. When livestock is not closely monitored but is reliant on human-maintained feed and water sources, lack of husbandry may also cause harm when emaciation and perishing occur [36].

#### 3.1.2. Livestock Transport

Transport of livestock causes stress and injury, whether livestock is transported via road [37], sea [38], or air [39]. The severity of animal welfare impacts arising from transport is related to the duration of the journey (e.g., three-week sea journeys for Australian sheep [40]), and stressors such as heat [41], overcrowding (stocking density [42]), vehicle motion [43] and transport of juvenile animals (e.g., dairy calves [44]). These harms are relatively well described in animal welfare science, having been the subject of several decades of research scrutiny [42].

#### 3.1.3. Working Animals Used in Farming and Hunting

Type 1 food production harms also encompass negative impacts on those domestic or captive animals not intended for consumption. This group encompasses working dogs (“sheep dogs”) [45], livestock guardian animals [46], and “beasts of burden” (e.g., stock horses or Asian elephants (*Elephas maximus*)) used to plough, till, or transport livestock or agricultural products [47]. More obscure uses include the use of captive raptors (“falconry”) to deter aggregations of wild birds that negatively impact crop fields [48]. Type 1 harms also extend to working animals used in hunting, such as the use of dogs for feral pig (*Sus scrofa*) hunting in Australia [49] or black bear (*Ursus americanus*) hunting in the USA [50]. Dogs are also commonly used to detect and retrieve hunted animals in developing nations, such as subsistence hunters that rely on dogs to amplify hunting success in Papua New Guinea [51]. There are important animal welfare impacts to consider for domestic animals (usually dogs) used in hunting for subsistence or commercial harvesting [49]. These impacts include the risk of exhaustion, heat stress, attacks from wild animals [50], vehicular trauma, snake bite, contracting infectious diseases from hunted animals [52], misadventure (e.g., becoming lost), and accidentally being shot [53].

### 3.2. Type 2 Harms

#### 3.2.1. Livestock Slaughter

All livestock are ultimately slaughtered for the production of meat, leather, or other products. The manner in which they are killed, and prepared for killing, create welfare impacts. Vast numbers of animals are killed every year for this purpose: estimated at 50 billion chickens, 1.4 billion pigs, 1 billion sheep and goats, 1.2 billion rabbits and 0.3 billion cattle [14]. There is abundant literature detailing the animal welfare impacts associated with livestock slaughter, including stress due to confinement, slaughter methods that do not induce immediate insensibility (e.g., Halal [54]), and failure of methods to produce immediate insensibility (e.g., misplaced captive bolt shots [55]).

#### 3.2.2. Wildlife Harvesting

Many species of wildlife are harvested for food production globally which continues to be an important source of protein for humans worldwide [56]. When wild species are killed and the meat sold for economic gain, this is termed commercial harvesting. A wide variety of birds, reptiles and mammals are commonly harvested, although the most widely harvested wildlife populations are aquatic and marine fishes. The magnitude of Type 2 harms imposed by fishing are enormous, with estimates of 1–2 trillion fish killed annually worldwide, excluding by-catch and discards [57]. Intentional harms imposed on wild fishes through fishing are imposed through the use of nets, lines, hooks, gaffes, etc. [58]. This includes impacts exerted through recreational fishing (angling) [59]. Marine mammals are also harvested, but to a much lesser extent, and harvesting methods differ from those applied to fish. Harpooning [60] is used for whales, “drive hunts” are used for dolphins [61], and blunt trauma (“clubbing”) for some ice-breeding species such as harp seals (*Pagophilus groenlandicus*) [62]. 

Professional consumptive use of terrestrial wildlife typically involves in situ killing of hyperabundant large herbivores, particularly ungulates and marsupials. Prominent examples include impala (*Aepyceros melampus*) harvesting in southern Africa [63] and kangaroo and wallaby (*Osphranter* and *Macropus* spp.) harvesting in Australia [64]. Shooting is the most commonly used method to harvest terrestrial species, but trapping is also utilized [65]. Shot animals can suffer if they are not rendered immediately insensible via shooting or are non-fatally wounded [66]. The shooting of adult wild animals can also lead to orphaning of dependent juvenile animals [67], which can be minimized by deliberately killing juvenile animals as a priority [68]. 

Aside from the professional consumptive wildlife use described above, at least two other forms of wildlife harvesting are worth mentioning. First, recreational, subsistence or traditional hunting (as is typically practiced by Indigenous peoples) involve in situ killing of wildlife at a smaller scale. Access to these food products is generally restricted to local populations. Recreational hunting is applied to a wide range of animal species with a variety of methods (e.g., shotguns, dogs, archery, rifles, traps, snares, etc.), and traditional hunting often employs yet other methods, such as physical capture from boats for sea turtles and dugongs (*Dugong dugon*) [69]. Second, the live-capture of wild animals for sale in live-animal markets or “wet markets” (ex situ killing) also occurs in many tropical Asian countries [70]. It is beyond the scope of this paper to explore these two types of wildlife harvesting in detail but they are described in conservation literature [71].

#### 3.2.3. Wildlife Damage Management

##### Wild Herbivore and Omnivore Control

Conflict is common between grazing herbivores or omnivores and cropping or horticulture [72]. Herbivorous wildlife (ether native or invasive animals) are commonly harmed through the application of lethal and non-lethal mitigation efforts. For example, wild pigs/wild boar (*Sus scrofa*) in the USA are often culled to reduce crop loss [73]. African savanna elephants (*Loxodonta africana*) are often harassed or killed in southern Africa and southern Asia when they attempt to raid crops [74]. Primates are similarly persecuted in the same regions when they engage in crop-raiding [75]. Wild birds are sometimes culled to protect crops that they may damage, e.g., grapes or rice [76]. In other settings, non-lethal deterrents (auditory and chemical) are used to prevent damage to crops by migratory bird species such as Canada geese (*Branta canadensis*) [77]. Large mammalian grazers (e.g., macropods) are also commonly culled or excluded to reduce competition with livestock, thereby reducing total grazing pressure, on extensive grazing properties [78].

Wild herbivores are also culled or harassed to prevent damage to agricultural infrastructure. This occurs, for example, to prevent damage to silo bags by armadillos (*Chaetophractus villosus*) on grain farms (e.g., in Argentina) [79], or to prevent damage to fences and water points by feral camels (*Camelus dromedarius*) on pastoral cattle properties in arid Australia [80]. 

Many frugivorous species of mammals and birds are culled, excluded, or harassed to prevent damage to fruit crops. Examples include culling flying foxes (*Pteropus niger*) to protect mango and lychee orchards in tropical and semi-tropical countries such as Mauritius [81]. Extreme examples of horticultural protection measures targeting bats include the construction of electrified grids over Australian orchards designed to electrocute flying foxes [82].

##### Wild Predator Control

Wild predators that depredate (or are perceived to depredate) livestock (Section 3.4.4) are often subjected to control measures worldwide. Various methods are used to reduce the frequency of predation events, and range from lethal means such as poison baiting, trapping and shooting, to non-lethal means such as deterrents, fencing and fladry [13]. Marine predators, notably pinnipeds, are also occasionally culled as predators of commercially important fishes in the belief that fish consumption by marine mammals represents losses that would otherwise be available to fisheries. Culling marine mammals ostensibly to protect fish stocks has a long history and has been undertaken in many parts of the world, such as the culling of grey seals (*Halichoerus grypus*) in the Baltic Sea to protect Atlantic cod (*Gadus morhua*) [83].

##### Rodent Control

Rodent control is another example of a Type 2 harm that is particularly common in grain production (cropping) systems to reduce grain loss [84,85]. It is also common in residential homes and similar settings to protect personal food stores. Anticoagulant poisons are most frequently used to kill large numbers of rodents [86], especially during plague conditions, though a variety of toxins are available. The scale at which rodents are killed for crop production in some countries is considerable. During mouse plagues in Australian croplands, conservative estimates suggest at least 100 mice are killed per hectare per year to grow grain [84]. Approximately 2.6 million rats were physically collected and killed in Myanmar over a 3-month period to protect rice crops and alleviate human starvation following a rodent outbreak linked to Cyclone Nargis [87].

##### Parasite and Infectious Disease Control 

Culling of wild animals is often undertaken to prevent the transmission of infectious diseases to livestock. Examples include culling of brushtail possums (*Trichosurus vulpecula*) to reduce transmission of tuberculosis to cattle in New Zealand [88], culling of badgers (*Meles meles*) for the same reason in the United Kingdom [89], or culling of bison (*Bison bison*) to reduce transmission of brucellosis to cattle in the USA [90]. A few interesting practices exist whereby sentient parasites are deliberately harmed to protect livestock. One example was the practice of lethal control of vampire bats (*Desmodus rotundus*, *Diaemus youngii* and *Diphylla ecaudata*) by systemic treatment of livestock with anticoagulant chemicals in Mexico in past decades, in an attempt to prevent cattle from contracting rabies [91]. 

#### 3.2.4. Food Safety Testing

Experimental (laboratory) animal testing is used extensively for food safety testing. This is a relatively poorly described Type 2 harm associated with food production and it has been suggested that the general public is not aware of the extent of animal experimentation carried out to ensure the safety of human food products [92]. It is estimated that about the same number of animals are used for the testing of food additives for humans as for cosmetics [92]. This form of harm is most commonly associated with processed foods, e.g., cereal grain products such as breakfast cereal, processed meat, and products containing dried dairy products. The testing of crop protection products (i.e., fungicides, herbicides, insecticides) makes up ~10% of all toxicological animal use. As a result, these harms are applied most consistently to those food systems that rely on the processing of foodstuffs and the use of additives, as opposed to foods that are relatively unprocessed. Animal welfare impacts imposed on these laboratory animals encompass all of the harms typically associated with experimental animals [93].

### 3.3. Type 3 Harms

#### 3.3.1. Land Clearing

Land clearing for agriculture is a recognized threat to biodiversity through the loss of wildlife habitat. While the once-off population-level effects on ecosystems are obvious, the ongoing welfare impacts at an individual animal level are also enormous, constituting an influential Type 3 harm [21]. The mechanism via which land clearing threatens wildlife populations is through death, displacement and local resource depletion for wild animals that reside in forests or woodlands that are cleared [94]. The clearing of native vegetation and its replacement with agricultural land use causes the death and extirpation of the majority of local extant vertebrates and invertebrates [94]. Land clearing for agriculture has occurred for millennia and is ongoing, not only in developing countries such as Indonesia [95] but also in post-industrial countries that rely heavily on agriculture, such as Australia [94]. Prominent examples of this process include land clearing to facilitate beef grazing [96] and the growing of soybeans in Brazil [97] and displacement of wildlife, such as orangutans by palm oil plantations in south-east Asia [18].

#### 3.3.2. Tilling, Ploughing and Harvesting of Cropping Land

Annual crops require replanting every year which is typically associated with tilling fields in traditional cropping systems. Tilling may lead to direct deaths and injuries for soil- and field-dwelling animals inhabiting agricultural land, such as rodents [85]. Small mammals can reach densities of more than 100 individuals per hectare on agricultural land [98] and many or most of these animals are killed or otherwise affected by ploughing and harvesting [99,100,101]. Common voles (*Microtus arvalis*) experience extensive mortality following tilling events in European cropping fields [99]. Polynesian rats (*Rattus exulans*) are often killed by equipment or suffocated in their burrows under compacted soil during the mechanical harvesting of sugar cane in Hawaii [102]. Many radio-tagged wood mice (*Apodemus sylvaticus*) also disappeared within a week because of predation after protective vegetation was removed during grain harvesting in the United Kingdom [103]. Given the estimated 1.4 billion hectares of arable land in the world [14], routine soil preparation practices in cropping systems affect the welfare of an enormous number of animals.

Mechanical equipment used to harvest grain crops may also inadvertently injure wildlife that inhabit cultivated fields, including combine injuries that may happen when deer conceal fawns in fields to limit predation. In Italy, one study found that combine harvesters are a major cause of injury for newborn roe deer (*Capreolus capreolus*) concealed in crops [104]. The majority of these animals were euthanized due to the severity of the lacerations suffered when caught in these powerful machines. Smaller mammals may also be injured or killed by mechanical harvesters, including common hamsters (*Cricetus cricetus*) and wood mice [103]. Cutting hay can also cause injury and death to ground-nesting birds such as bobolinks (*Dolichonyx oryzivorus*) [105] and ring-necked pheasants (*Phasianus colchicus*) [106] in the USA. 

#### 3.3.3. Entanglement

Wild animals can become entangled in a variety of anthropogenic structures used for food production and protection, with lethal or non-lethal effects. This category of harms applies to fencing, netting, and marine debris.

##### Fencing

**Figure 1:**
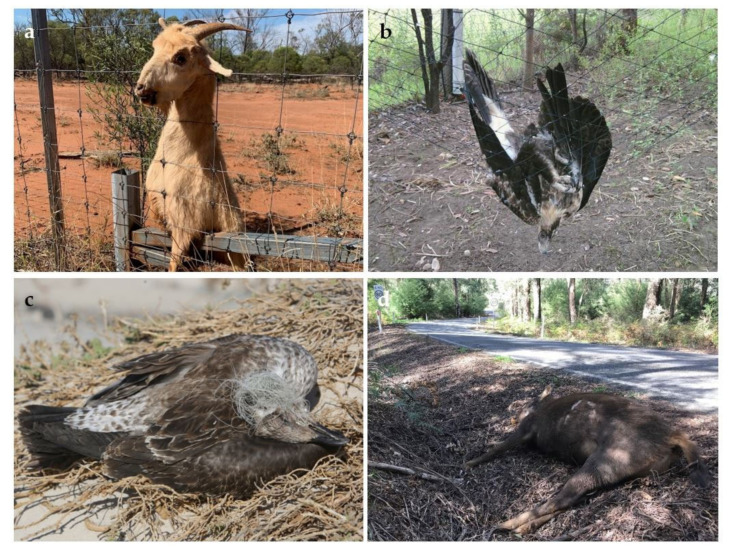
Examples of Type 3 harms affecting wild animals as a result of food production: (**a**) A feral goat (*Capra hircus*) caught and killed in livestock fencing; (**b**) A wedge-tailed eagle (*Aquila audax*) caught in horticultural netting; (**c**) A kelp gull (*Larus dominicanus*) entangled in fishing line; (**d**) A sambar deer (*Rusa unicolor*) struck and killed by a truck used for food transportation. (Photos: Benjamin Allen, Gary Williams, Peter Ryan, Jordan Hampton).

Fencing can be used to reduce harms caused by wildlife interacting with agricultural infrastructure, consuming plant produce or competing with livestock. Fencing is often used to keep livestock in paddocks and to exclude wild animals (and people) from areas where food crops and livestock are grown. However, there are a variety of harmful side-effects to fencing [107], encompassing several pathways for harm [108]. A robust body of literature on movement and crossing behaviors around fences shows the physiological and fitness risks that fences can impose as animals search for breaks [109], alter their optimal movement or foraging patterns [110], adopt novel crossing behaviors or are injured or killed in efforts to cross [111]. There are many examples of studies documenting negative animal welfare impacts of fencing [112,113,114,115]. Fencing imposes direct harms on wildlife accidentally caught or entangled in fences (Figure 1a), whereby susceptible species regularly die or require rescue [116].

To take examples from two continents, species susceptible to fence entanglement include pronghorn (*Antilocapra americana*), mule deer (*Odocoileus hemionus*), and elk (*Cervus elaphus*) in North America [111], and kangaroos, introduced ungulates (Figure 1a) and emus (*Dromaius novaehollandiae*) in Australia [117]. Fences also inhibit the movement and migration of many large terrestrial wildlife species, excluding them from important resources (e.g., water points) and altering natural behavior [13]. Less direct impacts of fencing have also been observed.

Electric fencing has the additional risk of causing animal mortality through electrocution. A South African study found that wildlife species such as leopard tortoises (*Stigmochelys pardalis*) and pangolins (*Manis temminckii*)—which remain stationary in response to electrocution [118], as opposed to fleeing—are frequently killed by electric fences [119]. Even plain wire and barbed-wire fences used to contain livestock are known to cause welfare harms to livestock and wildlife, which have been debated for decades [120].

##### Netting

Netting can be used to exclude wildlife (particularly volant (flying) species) from sensitive agricultural produce, especially horticulture. Netting is commonly used over orchards and vineyards for this reason. Many frugivorous terrestrial wildlife species commonly become entangled in netting used to protect orchards, including bats [121], birds (Figure 1b) [122], and snakes [123]. Entangled animals may be killed outright, attacked by predators while immobile, or euthanased or rehabilitated by humans.

##### Marine Debris and Plastic Waste

Marine debris can also cause entanglement for oceanic species of wildlife. It is estimated that more than 141 million tons of plastic are used each year as food packaging [124], making a major contribution to the estimated 250 thousand tons of marine debris currently afloat in the world’s oceans [125]. The scale of this issue can be visualized through modern developments such as the discovery of the Great Pacific Garbage Patch [126]. The ecological risk posed by discarded plastic has been intensively studied in the past decade and is now broadly understood by the public [127]. Individual animals are harmed by ingesting plastic packaging [128,129] and by becoming entangled in plastic packaging. A familiar and vivid example is that of water birds and turtles entangled in six-pack beverage holders [130]. Commercial and recreational fishing also results in the creation of marine debris, e.g., discarded fishing lines, nets, buoys, etc. Entanglement in debris is a common cause of mortality and injury for many marine and coastal species [131], including whales [132] and seabirds such as Australian pelicans (*Pelecanus conspicillatus*) [121] and kelp gulls (*Larus dominicanus*) (Figure 1c). Impacts also include animals dying from ingesting marine debris, which is a common finding for a variety of seabird species from Australia and New Zealand [133].

#### 3.3.4. Damming of Water Bodies for Irrigation

Several types of intensive agriculture rely on irrigation, particularly when water-thirsty crops are grown in semi-arid areas. Well-known examples include growing vegetables in Israel, rice in inland Australia, or almonds in the USA [134]. Intensive livestock production also sometimes relies on irrigation, particularly in the case of pasture-driven dairy farms established in arid areas. Irrigation also impacts wildlife if water is drawn from underground aquifers rather than from damming of above-ground watercourses. Such impacts are being noticed in parts of the world that have used groundwater unsustainably, such as the south-west of the USA [135]. Although enabling considerable increases in productivity or the quantity of food grown per unit of land area, irrigation often requires the damming of natural waterways, and the ever-rising demand for water to support irrigated agriculture has led to the demise of wetlands and their associated wildlife for decades [136].

The wild animals that have been most obviously affected by this process are waterbirds, but also include amphibians, fish, aquatic mammals and invertebrates. The global thirst for water is so pervasive that many wetlands considered to be hemispheric reserves for waterbirds have been heavily affected, for example, the Aral Sea in central Asia [137]. In the Murrumbidgee valley of Australia, waterbird numbers estimated during annual aerial surveys collapsed by 90% over 19 years due to damming of rivers for irrigation [138]. Damming also inhibits the migration of fish to and from spawning grounds.

#### 3.3.5. Transport Effects

Transport of food can cause harm to wild animals through the impacts of roads disrupting vertebrate wildlife species through habitat fragmentation and collisions with vehicles (“animal-vehicle collisions”; AVCs) (Figure 1d) [139]. Any vehicles used in the transportation of food or livestock have the potential to collide with, and harm (often killing) wild animals. This is true of land-based road vehicles [140], ships [141], and aircraft (“bird strike”) [142]. Risks are heightened for vehicles carrying food products given wildlife may also be attracted to energy-rich food accidentally spilled or discarded, such as grizzly bears (*Ursus arctos horribilis*) attracted to grain-carrying trains in Canada [143]. While many readers will have first-hand experience of striking a wild animal in a car, the extent of such collisions in aquatic and marine environments may be less obvious. The provocatively titled review article “How we all kill whales” provides an insight into the extent of impacts from fishing and ship transport on world cetacean populations [141]. Transportation corridors can also attract wildlife via habitat enhancement (e.g., moose (*Alces alces*) attracted to the near-road areas by de-icing salts that accumulate in pools at snowmelt [144]) and foraging opportunities (e.g., corvids and raptors focusing their foraging near roads [145]), and thus exacerbate AVC risks [143]. Aside from AVCs, food transport vehicles that generate loud noise may also disturb the movements of wild animals, as has been demonstrated for aircraft [146].

### 3.4. Type 4 Harms

#### 3.4.1. Pollution

The broad term “pollution” relates to the introduction of harmful materials into the environment. Types of pollution relevant to food production include chemical pollution, noise, and light pollution. Modern anthropogenic climate change is also a product of special types of pollution, but we will consider this separately in Section 3.4.2.

##### Eutrophication

**Figure 2:**
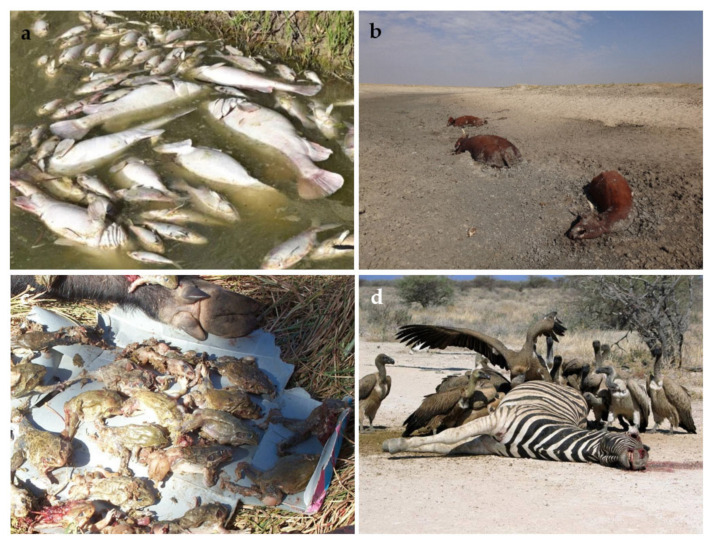
Examples of type 4 harms affecting animals as a result of food production: (**a**) Mass mortality of freshwater fish caused by eutrophication; (**b**) Free-ranging cattle (*Bos taurus*) that have perished during a drought influenced by climate change; (**c**) Mortality of frogs through predation by an invasive species, feral pigs (*Sus scrofa*), whose populations are derived from escaped agricultural animals; (**d**) mortality of a plains zebra (*Equus quagga*) from an infectious disease, anthrax, introduced via livestock. (Photos: Jonathon Howard, Jordan Hampton, Jim Mitchell, Wayne Getz).

There are several forms of chemical pollution, whereby a wide range of anthropogenic activities lead to the deposition of harmful chemicals into the environment. Eutrophication is a specific type of pollution that occurs when a body of water becomes overly enriched with minerals and nutrients from the discharge of soil nutrients from the environment [147]. Exposure of freshwater fish to agricultural ammonia affects their growth and changes their energy conversion efficiency [148]. It is particularly associated with agricultural activities that utilize fertilizers (e.g., cropping) [149] and results in wide-ranging and undesirable changes in coastal ecosystems [150]. Eutrophication can lead to hypoxia (“dead zones”) [151] and has caused mass fish kills (Figure 2a) and decreases in other aquatic life in numerous sites from around the world [152]. For succinctness, we will include in this category the effects of nitrogen-rich effluent from intensive animal systems such as dairy farming [153], which can cause profound ecological changes to rivers receiving large volumes [154].

##### Insecticides and Pesticides

Agricultural insecticides also have profound impacts on wildlife, such as vultures. Bird mortalities may also occur due to feeding on seeds treated with insecticides or other pesticides. This phenomenon has been observed around the world [22], from the USA [155] to India [156] and Korea [157]. These risks have been widely understood by the public since Rachel Carson’s 1962 book “Silent Spring” [158] but continue to the present day, and there is still concern that toxicity knowledge is incomplete for modern poisons such as glyphosate [159]. Exposure to organochlorines and other pesticides is considered to be the most important threat currently affecting vultures worldwide, through both accidental and deliberate abuse. Non-lethal exposure to these compounds occurs on every continent that vultures inhabit [160]. Poisoning of wild birds through these chemicals is not limited to scavengers, with wildlife that feeds on crops or invertebrates sprayed with insecticides commonly negatively affected. For example, paralysis syndromes occur in frugivorous birds affected by organophosphates in fruit-growing areas [161].

##### Secondary Poisoning

Secondary poisoning or “relay toxicity” is a type of chemical pollution specific to the indirect effects on non-target wildlife species that consume the body of another type of wild animal poisoned intentionally. Typical examples affect wild predators that consume smaller animals deliberately poisoned by humans. For example, cases of non-target anticoagulant poisoning are common in birds of prey from residues in rodents killed to protect stored grain [162].

##### Pharmaceutical Compounds

Veterinary pharmaceuticals (e.g., antibiotics) used to treat livestock, working animals or farmed fish may become available to wildlife and cause deleterious effects. These compounds are known to be widespread environmental contaminants but knowledge of exposure patterns and possible effects in wildlife remain poorly characterized [163]. One prominent recent example resulting from food production is the indirect poisoning of vultures (*Gyps* spp.) feeding on livestock carcasses with diclofenac; an anti-inflammatory drug that is used commonly to treat cattle on the Indian sub-continent [164].

##### Noise Pollution

Noise pollution is a more subtle form of pollution that can nonetheless cause profound disturbance to wild and domestic animals [146]. The magnitude and spread of anthropogenic noise pollution are often much greater than those of natural noise and have been shown to have a range of harmful effects on wildlife [165]. Noisy human activities may influence the physiology and behavior of wildlife living in adjoining areas [166] and these effects have been recognized since at least 1978 [167]. This phenomenon has been demonstrated for cetaceans [168], fish [169], terrestrial mammals [170], birds [171] and zoo animals [172], among others.

Noises generated in food production clearly fit into this category of harms and may cause intentional or unintentional harm to animals. Intentional noise is a Type 2 harm and includes sources such as gas guns used to deter birds from damaging orchards. For example, gas guns are commonly used to deter Baudin’s cockatoos (*Calyptorhynchus baudinii*) from orchards in the south-west of Australia [173]. Even more animals are affected by noise that is not intended to harm (Type 4 harms). For example, livestock road transport contributes to traffic noise [170] while shipping of agricultural produce or live animals contributes to ocean noise [168]. Movement patterns of female white-tailed deer (*Odocoileus virginianus*) have also been affected by intensive row-crop agriculture in the USA, but quieter times (e.g., crop emergence and harvest) had minimal effects [174]. Gunfire is another source of noise disturbance to wildlife [166] and is associated with all wildlife harvesting methods that use shooting, although the magnitude of this effect may be minimized by the use of suppressors on rifles [175].

##### Light Pollution

Light pollution is yet another form of harmful contamination from anthropogenic activities. Artificial light at night (ALAN) refers to nocturnal anthropogenic light sources which may have profound effects on animals that are highly sensitive to natural lunar light levels [176]. Daily, lunar, and seasonal cycles of natural light have been key forms of environmental variation across the Earth’s surface since the first emergence of life. However, the natural patterns of light over the last century have been greatly disrupted through the introduction of ALAN, causing profound changes to the behavior of many wild species [177], as well as domestic animals. For example, artificial lighting is used to facilitate egg production in layer chickens and high-intensity lights and blue-lighting have been associated with increased incidences of feather pecking and aggressive behavior [178]. Any food production industry that relies on widespread ALAN will induce negative effects, such as a reduction in the ecosystem services of frugivorous bats [177].

##### Miscellaneous

A miscellaneous type of pollution is commonly associated with shooting: exposure of scavenging animals to toxic lead (Pb) fragments from lead-based bullets. This process has been shown to cause toxic lead exposure for taxa such as birds of prey [179] and mammals such as Tasmanian devils (*Sarcophilus harrisii*) [180] that frequently scavenge shot carcasses. This harm is increasingly being avoided by using lead-free bullets [181].

#### 3.4.2. Greenhouse Gasses and Climate Change

The ways in which climate change may impact animal welfare are numerous and profound but are not always intuitive [182]. These impacts are already being observed for many wildlife and livestock species (Figure 2b) [183]. While some effects are relatively obvious, including the mega-fires in south-eastern Australia in 2019–2020 [184] and mass mortality events in bats during extreme heat events [185], other effects are less obvious. For example, a long-term study in Gabon showed an 11% decline in the body condition of fruit-dependent African forest elephants (*Loxodonta cyclotis*) associated with a sharp decline in fruiting as a result of climate change [186]. Greater one-horned rhinoceroses (*Rhinoceros unicornis*) in the Terai region of India and Nepal are also adversely affected by intensified flooding and habitat loss associated with climate change [187]. It is almost certain that future studies will reveal more and more ways in which human-induced climate change is negatively affecting the welfare of wild and domestic animals around the world [182].

All food production systems produce greenhouse gas emissions (GHGEs) to some degree [188] and agricultural activity is a significant source of GHGEs [189], producing in the order of one-third of the world’s emissions [190]. However, it should be noted that some plant farming systems can have the opposite effect through carbon sequestration [191]. Carbon dioxide (CO_2_) is produced by a wide range of human activities associated with agriculture, ranging from electricity production to power equipment and refrigeration, to the burning of fossil fuels for transport and distribution of food. The carbon footprints of many food types have been studied extensively in the interest of sustainability [192]. Other greenhouse gases produced by food systems may also contribute to climate change and several other GHGEs, that are more potent than CO_2_, are released in huge quantities by food production. Nitrous oxide (N_2_O) is an especially potent greenhouse gas that is created concurrently with CO_2_ and methane (CH_4_) during fertilizer production [190]. Fertilized croplands also emit N_2_O [189]. Production of methane (CH_4_) is a ubiquitous, apparently unavoidable side effect of fermentative fiber digestion by symbiotic microbiota in mammalian herbivores. Relatively large quantities of methane are produced by all ruminants (e.g., cattle and sheep) through eructation [193] but not by other meat-producing animals such as marsupial herbivores (e.g., kangaroos) [194]. The production of GHGEs is particularly high for food products that require fertilizer use, refrigeration and long-distance transport from where they are grown to where they are consumed [5].

Other sources of GHGEs from food production are less obvious. For example, agricultural crop residue burning, as commonly practiced in countries such as India [195], contributes towards GHGEs. Even for food sources traditionally considered to have minimal GHGEs, impacts still exist. For example, the long driving distances covered by Italian recreational hunters to harvest wild red deer (*Cervus elaphus*) represent almost 85% of GHGEs for this hunting method [188]. Some analyses have yielded surprising results; for example, the GHGEs associated with intensive cattle production (finished in feedlots with growth-enhancing technology) are lower than that of extensive (grass-fed) cattle production systems [192].

It is also worth noting that energy-intensive food production systems will harm animals even if GHGEs are avoided through the use of renewable energy; wind farms cause wild bird and bat injuries and mortalities [196], as do solar arrays [197], and tidal energy schemes impact marine animals [198].

#### 3.4.3. Introduction of Invasive Species

Some farmed animals have the potential to become invasive species that cause subsequent harm to native species. Common livestock examples include pigs (Figure 2b), goats (*Capra hircus*) and fallow deer (*Dama dama*) [199], and lesser-known examples include introduced bee species that escape from apiaries. For example, European honey bees (*Apis mellifera*) are used for commercial honey production in many places including Australia, where they represent a source of competition for floral resources with native nectar and pollen feeding insects such as the native Australian bee *Hylaeus alcyoneus* [200]. Invasive bees harm animals through stinging and competition with wild animals for nest hollows, as shown in the case of honey bees excluding the endangered Lear’s macaw (*Anodorhynchus leari*) from preferred nesting hollows in Brazil [201]. Historical examples of invasive species that have been established due to agriculture or agricultural support activities include cane toads (*Rhinella marina*), imported to Australia to provide biological control of cane beetles (*Dermolepida albohirtum*) in sugar cane crops. Cane toads have since contributed to declines in a variety of native reptile species across large swaths of eastern and northern Australia [202].

Invasive plants introduced as horticultural or fodder species may also become invasive weeds and harm animals through modifications to environments. A prominent example is buffel grass (*Cenchrus ciliaris*), a globally significant invader now widespread across central Australia that was originally introduced as forage for rangeland cattle. Buffel infestation has widespread impacts on native animals in Australia, including reductions in floral diversity and increases in the intensity and frequency of wildfires that negatively affect faunal diversity [203]. Transport effects (Section 3.3.5) act synergistically with invasive species introduction risks when it comes to sea transport of food products, which facilitates the introduction of invasive marine species from ballast water [204].

#### 3.4.4. Predation of Domestic Animals

Related to the effects of wild invasive species is the special case of predation that occurs when introduced species are kept as livestock or used as working animals [205]. In the same way that predation from pet cats is considered a Type 4 harm for wild birds (Section 2.2), Type 4 harms due to predation from large wild mammalian predators occur when livestock are kept in conditions in which they are not closely monitored (e.g., extensive and pastoral systems) [13]. Most of these predators are canids, felids or ursids, and well-known examples include grey wolves (*Canis lupus*) attacking cattle in the USA [206], dingoes attacking sheep and cattle in Australia [13], leopards (*Panthera pardus*) attacking goats in southern Africa [207], and snow leopards (*Uncia uncia*) attacking livestock in mountainous areas of central Asia [208]. The same process may affect farmed fish that cannot escape a predator that enters a pen, for example, Australian fur seals (*Arctocephalus pusillus doriferus*) feeding on farmed Atlantic salmon (*Salmo salar*) in Tasmania, Australia [209]. Manipulation of wild predator populations may also result in negative impacts for native prey species. For example, kangaroo populations released from dingo suppression following dingo control to protect livestock can grow to grossly unsustainable numbers, resulting in mass death by starvation during times of drought [64].

#### 3.4.5. Exposure to Infectious Diseases

The animal welfare impacts of infectious diseases are not well documented [71] but can be profound [210]. Food production can lead to infectious disease outbreaks that impact the welfare of vast numbers of wild and domestic animals [211]. Familiar examples affecting predominantly domestic animals have included foot and mouth disease outbreaks in the United Kingdom caused by the importation of infected livestock [212]. A prominent example affecting wild animals was the spectacular outbreak of rinderpest in southern Africa in the late 19th century, also caused by the importation of infected livestock [213]. Similar patterns are followed in the present when wild African herbivores die from anthrax spread by livestock (Figure 2c) [214]. Less well-known examples of impacts exerted on wild animals are the 1995 and 1998 mass mortalities of pilchards (*Sardinops sagax*) observed in southern Australia caused by the introduction of a herpesvirus from frozen fish fed to sea-caged southern bluefin tuna (*Thunnus thynnus*) [215]. Mortalities in pilchards were around 60%, which is equivalent to hundreds of millions of fish killed [216]. Consequently, there were measurable secondary impacts in piscivorous species including little penguins (*Eudyptula minor*), which experienced increased mortality rates and failed to breed due to food shortage [217].

#### 3.4.6. Salinity

Dryland salinity associated with clearing and cropping or grazing is noticeable in many parts of the world such as south-west Australia and is associated with severe impacts on biodiversity [218]. Food production systems that rely on land clearing (especially cropping) have contributed heavily to the development of dryland salinity. Closely related to salinization but more severe, desertification is a type of land degradation in drylands in which biological productivity is lost due to natural processes or induced by human activities whereby fertile areas become increasingly more arid. Human activities that contribute to desertification include the expansion and intensive use of agricultural lands, poor irrigation practices, deforestation, and overgrazing. These unsustainable land uses place enormous pressure on the land by altering its soil chemistry and hydrology. These impacts from unsustainable agriculture (particularly cropping but also overgrazing) are being observed worldwide in semi-arid regions as widespread as East Asia [219], West Africa [220], Australia [218], and the Middle East [221].

The ways in which salinity and desertification harm animals are numerous and sometimes subtle. Short-term single-species impacts are especially evident for waterbirds breeding in inland wetlands affected by salinization. One example comes from studies of the health and behavior of American avocet (*Recurvirostra americana*) chicks exposed to saline conditions. Birds raised in highly saline conditions exhibited increased activity, decreased feeding and preening, weight loss and dehydration [222]. Long-term multi-species impacts are less explicit and include examples such as the introduction of sheep to arid Australia leading to the conversion of large tracts of perennial grasslands into deserts dominated by unpalatable spinifex (*Triodia* spp.), causing the decline of some native mammals and favoring of others [223].

#### 3.4.7. Soil Erosion

Soil erosion occurs during the intensive use of agricultural land, particularly following deforestation. This process has become a major global environmental threat [224]. Soil erosion creates profound ecological changes that can harm terrestrial wildlife through altered vegetation communities and marked changes to water quality in marine and freshwater habitats [14]. Soil erosion plays a key role in contributing to eutrophication (Section 3.4.1). Land use practices particularly associated with soil erosion include cropping on sloped and marginal land [225], overgrazing by livestock [226], and the removal of protective vegetation through repeated tilling or harvesting of crop residues [14].

#### 3.4.8. Disposal of Food Waste

Food wasted by humans is often accessible to wildlife, harming individual wildlife animals and negatively affecting ecological processes and community dynamics [227]. Food waste that is available to wildlife may artificially support anthropogenically elevated abundance levels that cannot be sustained [11]. This process leads to increased inter- or intra-species aggression, increased incidence of infectious diseases, and starvation when waste-derived food supplies diminish. A familiar example is that of seabirds that become reliant on refuse tips (garbage dumps), and subsequently suffer breeding failures when the supply of refuse is made unavailable [228]. This effect may be most pronounced in fisheries, where annually, it is estimated that >10 million tons of fish are discarded. In the North Sea, this process may support <6 million seabirds [229], with profound effects on populations likely if supply is reduced or disrupted.

#### 3.4.9. Depletion of Natural Resources

Depletion of natural resources may occur through overharvesting of wild plants, seaweed, fungi or animals [230]. The scale of this process is enormous, with 72% of the species listed as threatened or near-threatened on the IUCN Red List of Threatened Species being overexploited in 2016 [230]. The ecological impacts of these depleted resources can be far-reaching and may be most pronounced for wild predators when prey animals are depleted. Abundant prey is required for the survival of large carnivores. When sufficient prey is unavailable, large carnivore populations will decline, sometimes becoming locally extinct [231]. These effects are observed in terrestrial and marine ecosystems. For example, overharvesting of fish can lead to negative impacts on seabirds [232] through loss of body condition and reduced reproduction. On land, leopard (*Panthera pardus*) abundances in the Congo Basin rainforest declined and diets shifted towards smaller prey species in regions near human settlements due to competition with bushmeat hunters [233]. Over-harvesting of wild plants, seaweed, fungi can also have profound negative effects on wild herbivores [234]. Type 4 harms related to resource consumption occur to some degree regardless of whether resources are harvested sustainably or in excess.

## 4. Summaries of Food Production Systems

We described a number of examples of the four broad categories of animal harms that may result from food production systems. Our analysis described three forms of Type 1 harms, four forms of Type 2 harms, five forms of Type 3 harms and nine forms of Type 4 harms. It is natural that the number of ways that animals may be negatively affected increases as we move from intentional and direct towards unintentional and indirect. In total, we identified at least 21 ways in which food production systems may harm animals. This list is undoubtedly incomplete, but we present it as a conceptual guide. We now move to brief summaries of the harms imposed by several types of food production, listed in order of those imposing fewer harms to those imposing the most. A summary of animal harms for each food production system is provided in Table 1. In this table, animal harms Types 1–4 are used as headings with the 21 sub-categories of harms (described in Section 3) used as sub-headings. We reiterate that the intensity of the animal harms is beyond the scope of this commentary and is not discussed here. The list is likewise not exhaustive, in terms of all possible food production systems, nor all types of harms. A qualitative summary, as per [26], is provided for harms broadly associated with each food production system in Table 1.

### 4.1. Harvesting of Wild Mushrooms, Plants and Seaweeds

Harms for harvesting of wild mushrooms, terrestrial plants or seaweeds are almost identical. Wild plant harvesting is perhaps the oldest form of human food production and a well-known contemporary example is blueberry (*Vaccinium corymbosum*) picking in North America [235]. No Type 1 or 2 harms are imposed. Land clearing is not required. Foods acquired this way are rarely processed so Type 3 harms related to food safety testing and food additives are minimal. Subtle Type 3 harms may occur through the disturbance of wild animals [146]. For example, resident white-tailed deer are occasionally flushed by mushroom hunters in forested areas of the USA, but they typically returned to their home ranges by the next morning [236]. Other Type 3 harms depend on packaging (plastic waste). Type 4 harms occur but are most likely to be substantial if resources are severely depleted. This has been demonstrated for overharvesting of seaweed and wild plants, whereby harmful competition with wild herbivores and fungivores for resources occurs. Type 4 harms associated with pollution, invasive species and infectious diseases are non-existent and food waste is minimal but those associated with climate change occur, with GHGEs produced by transport and refrigeration of produce. 

Mushroom farming, as opposed to wild harvest, may impose a suite of harms similar to horticulture (Section 4.8) although circular food production systems (use of organic waste as substrate) may mitigate some of these [237]. Likewise, seaweed farming, as opposed to wild harvest, is practiced in south-east Asia [238] and imposes similar harms to seaweed harvesting with the addition of disturbance to sea beds. 

### 4.2. Apiary

No Type 1 harms are associated with honey production, at least on the assumption that insects are not sentient. Honey is rarely processed so Type 2 harms related to food additives and food safety testing are minimal. Land clearing is not required. However, Type 2 and Type 3 harms have been documented in some places such as the USA, where conflict with omnivorous wildlife results in Type 2 harms through trapping of skunks (*Mephitis* spp. and *Spirogate gracilis*) [239] and Type 3 harms through fencing to exclude black bears (*Ursus americanus*) [240]. Other Type 3 harms depend on packaging (plastic waste). Type 4 harms associated with apiaries relate to the potential for exotic bee species to become invasive and the introduction of infectious diseases [241]. Type 4 harms are also associated with climate change and the GHGEs produced by the transport of produce, although refrigeration is not required. Other forms of pollution and food waste are minimal.

### 4.3. Terrestrial Wildlife Harvesting

For succinctness, this section includes commercial harvesting and other forms of hunting of land-based wildlife. Type 1 harms are absent unless domestic animals (usually dogs) are used to aid hunting. Type 2 harms are obvious from shooting or trapping wild animals. Type 3 harms are few and land clearing is not required. Game meat is rarely processed so Type 3 harms related to food additives and food safety testing are minimal. Other Type 3 harms depend on the use of packaging (plastic waste). One Type 4 harm commonly associated with shooting is lead poisoning of wild scavengers through ingestion of fragments from lead-based bullets. Another Type 4 pollution harm is caused by noise disturbance from gunfire, which may be minimized by the use of suppressors [175]. Hunting has also incentivized and resulted in the release of invasive species worldwide, e.g., red deer in New Zealand [242]. Type 4 harms are also associated with climate change and the GHGEs produced by the transport and refrigeration of meat. Food waste Type 4 harms occur when the abundance and behavior of wild scavengers are changed when large numbers of gut piles from carcasses are made available through field butchering [227]. Finally, Type 4 harms related to depletion of natural resources may be imposed if harvesting is poorly regulated and reduces the abundance of the target species below the desired level (i.e., overharvesting).

A quite different set of harms apply to the type of consumptive wildlife use typical of “wet markets”. In this context, Type 1 harms are considerable, applying to wild animals taken into captivity in often cramped and stressful conditions [70]. Type 2 harms apply to the slaughter of these animals by vendors or purchasers. Type 3 harms relate to injuries suffered by non-target wild animal species caught in snares and traps [243]. Type 4 harms include ecological effects if rare animals are targeted or adversely impacted as non-target species, and finally, the conspicuous threat of infectious disease spread to other animals (and people) through housing multiple live and stressed wild animals in close proximity to each other [244].

### 4.4. Marine and Aquatic Wildlife Harvesting

For succinctness, this section includes all commercial fishing, whaling and marine mammal harvesting. Small-scale subsistence harvesting of marine animals by Indigenous peoples is not focused upon here. Type 1 harms are absent except for a few specialized practices, e.g., the use of domesticated otters (*Lutra perspicillata*) to herd wild fish into nets in Bangladesh [245]. Type 2 harms are obvious from the use of nets, lines, harpoons (whales), clubs or shooting (seals) or drives and capture (dolphins) of wild marine or aquatic animals. However, there is controversy about whether all target species, such as crustaceans, meet commonly accepted criteria for sentience [246], and there is good reason to think that non-cephalopod mollusks are not sentient (Section 5.5). Seafood is often processed so Type 2 harms related to food additives and food safety testing apply. Type 3 harms commonly associated with fishing include entanglement of non-target species (“by-catch”) in nets or on lines, as well as entanglement in, and ingestion of, discarded fishing lines, floats, hooks, and sinkers. Land clearing is not required, although fishing methods that involve dredging may create similar harms through profound ecological disturbance to benthic (sea floor) communities. Scallop dredging is one marine harvesting method associated with considerable Type 3 harms through this process [247]

Type 4 harms are imposed through resource depletion and may be pronounced if harvesting is poorly regulated and reduces the abundance of the target species below the desired level (i.e., overfishing), and this has been an exceedingly common occurrence worldwide with commercial fishing [248]. Another Type 4 pollution harm imposed by nearly all methods is noise pollution from motorized boats. For the few marine mammal harvesting methods that use shooting, the same Type 4 harm caused by environmental pollution of shot carcasses with lead fragments applies as for terrestrial mammal shooting. Other Type 4 harms are associated with climate change and the GHGEs produced by transport, refrigeration and freezing of meat. Finally, Type 4 harms are pronounced through food waste; large amounts of fisheries waste have profound effects on wild scavengers.

### 4.5. Extensive (Free-Range) Egg Production

This category comprises the raising of domestic poultry under extensive outdoor conditions for the purpose of egg production on pasture, forage, or food waste products. Type 1 harms are associated with domestication, including effects on breeding, occasional husbandry procedures and infectious disease events. In most cases, Type 1 harms also include those associated with animal transport, usually by land or air. Type 2 harms include lethal control of bird predators (e.g., red foxes (*Vulpes vulpes*) in the United Kingdom [249]), and egg-laying birds being slaughtered for meat at the end of their lives. Here, we assume that all egg-laying animals are ultimately slaughtered for meat or to produce fertilizer and other “non-meat” products. Eggs are rarely processed so harms related to food additives and food safety testing are minimal. Type 3 harms are minimal and may be restricted to fencing (where used). The use of plastic packaging is rare. Land clearing, irrigation and fertilizer are not generally required. Type 4 harms include GHGEs produced by the transport of eggs, and infectious disease risks to native birds [250]. Food waste is negligible.

### 4.6. Rangeland Pastoralism

This type of livestock production typically involves free-ranging herbivores foraging on native unimproved (uncleared) vegetation over large geographical areas with minimal human husbandry [36]. As well as domesticated livestock species, large wild ungulates may be raised in an identical way, referred to as “wildlife ranching” and commonly practiced in southern Africa [251]. Type 1 harms are relatively few as livestock raised in this way are managed effectively as free-ranging animals. Occasional husbandry procedures are used and are often dissimilar to those used in other livestock production systems. These procedures are often specialized and may be considerably painful, such as branding and surgical “spaying” (ovariectomy) [252]. Transport harms may be less frequent than for other forms of livestock production (i.e., animals may only be transported once in their lifetimes: for slaughter or live export) but can be of much greater duration due to the remote locations of pastoral enterprises. Another Type 1 harm occasionally incurred relates to the use of guardian animals (usually domestic dogs) to deter wild predators. Type 2 harms apply to all wild herbivores and carnivores culled to reduce competition with, and predation of, livestock. Rangeland meat is often processed (i.e., to make mince or ground meat) so Type 2 harms related to food additives and food safety testing apply. Type 3 harms are relatively few. Land clearing is not required, although some pastoralists may clear land for this purpose.

Crops are not usually fed to raised animals, nor are they commonly treated with veterinary chemicals. Fencing is sometimes, but not always, used to keep livestock within designated management units. However, when fencing is used, it is typically barbed wire that can cause harm to wildlife attempting to cross under or over it [253]. Irrigation is not generally used, but plastic packaging of meat may be. Type 4 harms include the risks of predation, emaciation and perishing to the livestock in the absence of regular human monitoring [13]. Rangeland pastoralism has also made a notable contribution to the release of invasive species, with several damaging species having escaped from livestock paddocks, e.g., feral goats in semi-arid Australia [254]. Considerable GHGEs are produced, arising from CO_2_ emissions associated with long-distance transport (land, sea and sometimes, air) of live animals and meat, freezing or refrigeration of meat as well as methane emissions from the species raised, which are almost exclusively ruminants [194]. Fertilizer is not commonly used. Extensive livestock is the domestic animals most commonly predated on by wild predators and infectious diseases arising from livestock may affect wildlife. Overgrazing commonly occurs and contributes to soil erosion. Food waste effects may occur locally at abattoirs.

### 4.7. Dryland Cropping

Dryland cropping refers to all production of plant crops on dry land (i.e., not reliant on irrigation, discussed below). Dryland cropping is typically characterized by plant monocultures of grasses, such as wheat and corn, or legumes. The food products derived from dryland cropping are unusually diverse as processing is common (cereal grains are the main component of so-called “ultra-processed foods” [255]), making generalization difficult. Type 1 harms are not incurred. Type 2 harms may be largely avoided in environments in which wildlife damage to crops is minimal, but Type 2 harms can be considerable if local wildlife cause crop damage and are killed or harassed. Such Type 2 harms may include rodent poisoning, shooting of birds that feed on crops, and culling of mammals that damage crops. Another Type 2 harm often involved is food safety testing for grain products that undergo industrial processing (e.g., breakfast cereals) and have additives applied. Type 3 harms are important, including land clearing, fertilizer use, pesticide spraying and fencing. Some Type 3 harms depend on the use of packaging (plastic waste). Type 4 harms are associated with climate change and the GHGEs produced by mechanical sowing, harvesting, and spraying, as well as CO_2_ emissions from the transport of grain and stubble burning. In addition, fertilizer use leads to considerable production of the potent greenhouse gas N_2_O [189]. Cropping is the leading cause of the Type 4 processes of salinity and desertification around the world and soil erosion commonly occurs. Food waste occurs due to the spoilage of stored grains. 

### 4.8. Horticulture

This general title is used here to describe all forms of vegetable and fruit production, including orchards and vineyards. Type 1 harms are not generally incurred unless domesticated herbivores are used to constrain grass growth in orchards (e.g., “grazed orchards” in Europe) [256], domesticated dogs are used to deter herbivores such as white-tailed deer from orchards [257], or falconry is used to deter wild birds from vegetable fields [48]. Type 2 harms may be largely avoided in environments in which wildlife damage to fruit crops is minimal but encompass food safety testing applied to additives and chemicals used for those horticultural products that require industrial processing (e.g., soybeans destined for tofu). Type 2 harms can be considerable if local fruit-eating wildlife cause crop damage and culling is employed. Fruit and vegetable extracts and oils are often processed so Type 2 harms related to food additives and food safety testing apply. Type 3 harms are extensive. Land clearing is required (e.g., palm oil plantations), and fertilizer and pesticide use is common. Irrigation may be needed for the intensive production of water-intensive plant species. Type 3 harms also include ploughing and other mechanical field operations that impact wild sentient animals inhabiting soil. Another Type 3 harm relates to entanglement in nets used to exclude wild animals from orchards (Figure 1a, b) [230]. Type 4 harms are also associated with climate change and the GHGEs produced by mechanical irrigation and spraying, and transport and refrigeration of produce, and N_2_O emissions from the production and use of fertilizer. Horticultural species may also give rise to invasive plants (weeds) that exert Type 4 impacts [258] or may act as vectors for other invasive species (e.g., insect pests and plant diseases) [259]. Food waste is considerable due to spoilage with fresh produce. 

There are some notable exceptions to the suite of harms described above with some specialized forms of horticulture. Systems such as tropical agro-forestry may alleviate many Type 3 and Type 4 harms by not requiring complete land clearing, but allowing persistence of selected native shade trees [260]. 

### 4.9. Irrigated Cropping

Irrigated cropping refers to all production of plant crops where water is diverted from natural waterbodies to the site of domesticated plants. Forms include flood, channel, terrace, and drip irrigation. Irrigated cropping is typically characterized by plant monocultures of water-intensive species such as rice, vegetables, or fruit, as well as nuts such as almonds. Type 1 harms are not incurred. Some Type 2 harms may be largely avoided in environments in which wildlife damage to crops is minimal. On the other hand, Type 2 harms can be considerable if local wildlife cause crop damage, e.g., culling of ducks or rats over rice fields [261]. Other Type 2 harms are incurred when irrigated crops (e.g., soybeans) undergo industrial processing, have additives applied and require food safety testing. Type 3 harms are the most numerous for this form of food production, and include land clearing, damming and irrigation, fertilizer use, pesticide use and fencing. Some Type 3 harms depend on additives and packaging (plastic waste). Type 4 harms include GHGEs from mechanical sowing, harvesting, and spraying, N_2_O from the production and use of fertilizer, as well as CO_2_ emissions from the transport of grain. Fertilizer use in irrigated cropping also leads to considerable production of N_2_O [189]. Damming for agricultural irrigation has been one of the leading worldwide causes of loss of wetlands supporting waterbirds and many other examples of biodiversity [136] and food waste is associated with fresh produce spoilage.

### 4.10. Edible Insects

Insects, like vertebrate livestock, can be raised for human consumption and are eaten regularly by at least 2 billion people worldwide, with predictions for this number to increase in the near future [262]. Although edible insects can be wild-harvested just as vertebrate wildlife can, most such insects are raised as livestock in specialized insect farms where insects must be fed on another source of protein, often fish or livestock by-products. As such, almost all harms associated with raising edible insects are associated with what they are fed. Aside from these impacts associated with insect feeds, harms are few. Type 1 harms are generally thought to be absent given that insects do not meet traditional criteria for sentience (Section 5.5). Type 2 harms are limited given their mode of death is also not considered harmful due to their lack of sentience. Insects are typically killed via chilling and freezing [263]. Insects are rarely processed so Type 2 harms related to food additives and food safety testing are minimal. Type 3 harms are not obvious; insect farms occupy very little space, broad-scale land clearing is not required, and the use of plastic packaging is minimal. Type 4 harms may be imposed if farmed insects are introduced to an area, or accidentally escape and become invasive species [264]. Type 4 harms are also associated with climate change and the GHGEs produced by transport, refrigeration and freezing of insects, but food waste is negligible.

### 4.11. Cellular Agriculture

Cellular agriculture is the most contemporary (and largely hypothetical) form of food production examined here, so some additional background is provided. This form of food production involves growing animal-based foods (i.e., “cultured meat” or “clean meat”) from cell cultures, as opposed to harvesting animal products from the animals themselves [265]. As with edible insects, almost all harms associated with cellular agriculture are associated with what nutrient source is used [266], with one point of deviation related to the relatively high electricity requirement for this form of production. Meat grown in a laboratory is currently grown using nutrient broth originally derived from some form of traditional agriculture, e.g., bovine fetal serum [265]. Moreover, some of the early experimental work that led to the development of cellular agriculture was performed on laboratory animals. Companies attempting to produce cultured meat are actively working to end this reliance on animal-based cell cultures, and so it seems unlikely that they will still be using such cultures when the products are commercially viable and brought to market. Hence, Type 1 harms are incurred if domestic animal products are used for nutrient broth. Type 2 harms are restricted to those associated with sourcing the nutrient broth (i.e., livestock production or cropping) or any additives applied to meat (food safety testing). Type 3 harms, likewise, are restricted to those associated with the nutrient broth source and any plastic packaging used. Type 4 harms are also related to those associated with the nutrient broth source, but also include the CO_2_ emissions associated with an energy-intensive meat production process.

### 4.12. Aquaculture and Mariculture

This category includes the raising of fish in captivity for meat production in aquaculture or mariculture. For conciseness, we also include all farming of crustaceans [267] here. Familiar Type 1 harms are associated with captivity, including effects of breeding, deprivation of natural behavior, husbandry procedures and infectious disease events. Type 2 harms include the slaughter of fish and culling, exclusion, or translocation of wild predators (often seals). Type 2 harms also include the killing of other animals to produce the food required for farmed fish. These Type 2 harms can be amplified significantly depending on the aquaculture system, e.g., some semi-intensive and intensive aquaculture systems input 2–5 times more fish protein than is produced by the farmed fish [268]. As previously stated, there is controversy about whether all farmed species, such as crustaceans, meet commonly accepted criteria for sentience (Section 5.5). Type 2 harms related to food additives and food safety testing may apply if meat is processed. Type 3 harms relate to wildlife becoming entangled in nets or discarded debris, noise from motorized boats, ship strikes, and plastic packaging used for meat. Type 4 harms include CO_2_ emissions from motorized boats and refrigeration of produce. Fish farming has also made a notable contribution to the release of invasive species [269], e.g., carp (*Cyprinus carpio*) in Australia [270]. A particularly important Type 4 harm that applies to this form of food production is the introduction of infectious diseases that may affect other marine animals [215] and their predators. As for wild fisheries harvest, Type 4 harms may be pronounced through food waste.

### 4.13. Intensive Egg Production

This category includes the raising of domestic poultry under intensive indoor conditions for the purpose of egg production whereby animals are raised exclusively on crops rather than pasture or forage. As such, all harms associated with dryland cropping are also associated with this food system. Considerable Type 1 harms are imposed that are associated with domestication. Additional harms are associated with confinement to restrictive housing (“cage laying”) [31]. In most cases, Type 1 harms also include those associated with animal transport, usually by land or air. Type 2 harms include the mass culling of male birds and any wildlife control (e.g., rodent poisoning) associated with cropping used to feed intensively housed animals. If food additives are used in the industrial processing of poultry products, Type 2 harms associated with food safety testing apply. Type 3 harms are considerable and associated with cropping, land clearing, fertilizer use and fencing. Some Type 3 harms depend on additives and packaging (plastic waste). Indoor egg production occupies minimal land area, so Type 4 harms are mostly associated with reliance on cropping as a feed source for reared animals. Food waste effects may occur locally at abattoirs.

### 4.14. Extensive Livestock Grazing

Unlike rangeland pastoralism, extensive livestock grazing involves land clearing to farm the animal more productively. Type 1 harms are imposed on livestock through domestication, transport, and related husbandry practices. The extent of Type 1 harms will be minimized in production systems that incorporate slaughter on the property of origin and will be greatest for those systems that require multiple forms of transport (e.g., long-distance land transport plus sea transport for livestock destined for live export [271]). Type 2 harms apply to all wild herbivores and carnivores that are culled to reduce competition with, and predation of, livestock respectively. Type 2 harms also include the culling of wild species that act as predators, competitors for forage, parasites, or spread infectious diseases. If meat is processed (i.e., to make mince or ground meat), harms related to food additives and food safety testing apply. Type 3 harms include land clearing, fencing and the requirement for long-distance transport. Type 4 harms include pollution from effluent (manure) and veterinary pharmaceuticals. Contributions to greenhouse gases include transport, refrigeration, and freezing of meat, as well as ruminant eructation (methane). Several damaging invasive species have escaped from extensive livestock paddocks, e.g., fallow deer [272], livestock are often predated on by wild predators and infectious diseases arising from livestock often affect wildlife. Food waste effects may occur locally at abattoirs.

### 4.15. Intensive Livestock Production

This category includes the raising of domestic mammals or birds for the purpose of meat production in intensive indoor barns or feedlots where animals are raised exclusively on crops rather than pasture or forage. As such, all harms associated with dryland cropping are also associated with this food system. Considerable Type 1 harms are those associated with domestication, including effects of breeding, loss of natural behavior, confined conditions, regular husbandry procedures and frequent infectious disease events [273]. These Type 1 harms may be of a particularly intense nature for invasive farming systems that require force-feeding (e.g., foie-gras geese [39]). In most cases, Type 1 harms also include those associated with animal transport, usually by land. Few Type 2 harms are directly related to livestock facilities, with the exception of rodent and bird control [274], but more are related to cropping and any additives applied to meat (food safety testing). Few Type 3 harms are associated with livestock raising but those associated with cropping are considerable, encompassing land clearing, fertilizer use (eutrophication) and fencing. Some Type 3 harms depend on packaging (plastic waste). Indoor livestock farming occupies minimal land area, so Type 4 harms are associated with reliance on cropping as a feed source for reared animals. Type 4 harms include pollution from effluent (manure), and antibiotics are heavily used, leading to pharmaceutical pollution. Electricity use for lighting and heating in livestock barns contributes to greenhouse gasses, and methane emissions occur through eructation when ruminants are raised, and infectious diseases frequently arise from high-density animal environments. Food waste effects may occur locally at abattoirs.

### 4.16. Dairy

This category refers to the husbandry of domestic cattle, buffalo, goats, sheep, or camels (and occasionally other mammals) to extract milk. Some dairy systems rely exclusively on rain-fed pasture (grass), but most systems use grain to feed lactating animals to some extent. As such, all harms associated with dryland (and often irrigated) cropping are also associated. Extensive Type 1 harms are imposed. These include selective breeding, intensive husbandry, lameness, physiological stress associated with extended lactation, common surgical procedures, intensively managed reproduction, and calf removal. In most cases, this also includes the Type 1 harms associated with livestock transport, usually by land but sometimes by sea or air.

Type 2 harms include the familiar processes of slaughtering dairy animals for meat production after their use for milk production ends, and culling of male calves [44]. Here, we assume that all milk-producing animals are ultimately slaughtered for meat or other animal products (i.e., fertilizer). Type 2 harms also extend to wildlife control associated with protecting dairy herds from predation, grazing competition, or transmission of infectious diseases. In addition, Type 2 harms commonly include food safety testing applied to additives used in dairy products.

Type 3 harms are influenced by the fact that dairy farming occupies relatively little land area when compared to extensive livestock grazing but is invariably associated with reliance on cropping as a feed source for reared animals to some degree. Hence, Type 3 harms are considerable and associated with land clearing, cropping, irrigation, fencing and plastic packaging for dairy products. Type 4 harms include effluent from dairy farms contributing to pollution, as does antibiotic use. Dairy farming also makes a sizeable contribution to eutrophication through heavy fertilizer use. GHGEs produced by CO_2_ emissions from electric-powered milking systems, refrigeration and freezing, and motorized transport, N_2_O emissions from fertilizer and methane emissions from almost exclusively ruminant livestock. Dairy farming may have made occasional contributions to the release of invasive species (e.g., dairy goats [275]), dairy animals are sometimes subject to predation by wild predators, and infectious diseases from high-density farms may spread to wildlife populations. Food waste effects may occur locally at dairies and abattoirs.

## 5. Comparing and Ranking Harms

Table 1 makes clear that all current forms of food production harm animals in certain ways. Even vegan food products have (what may be to many) a surprisingly high harm footprint, largely because contemporary plant agriculture is intensive in terms of the land, water, chemicals and energy that it requires [276], affecting a multitude of wild animals in subtle and sometimes non-intuitive ways. These analyses placed no weight on what type of harm was imposed, its severity, or on which species it was imposed. These variables and animal welfare trade-offs all require consideration before meaningful comparisons can be made. So, the next question we need to ask is: how do we compare these dissimilar harms?

### 5.1. Conflicting Harms

From the preceding sections, it is apparent that some food production techniques avoid imposing one type of harm but subsequently incur a different type of harm. In many cases, particularly for non-animal-based food production systems, intentional harms are avoided by not producing animals, but harms are imposed on wildlife that are unintentionally affected by producing or harvesting plants, mushrooms and/or seaweed.

One example of trading off different types of harm can be seen in a comparison of professional terrestrial wildlife harvesting and dryland cropping, both of which may be realistic food-producing land uses for many semi-arid parts of the world [277]. Neither approach imposes Type 1 harms, avoiding the use of domestic or captive animals. Wildlife harvesting imposes obvious Type 2 harms to shot animals, while dryland cropping imposes Type 2 harms when rodents are killed to protect stored grain, wildlife feeding on crops are controlled or food safety testing is required for processed grain products. However, while wildlife harvesting imposes few Type 3 harms, those imposed by dryland copping are considerable, including land clearing, fencing, and use of fertilizer. Type 4 harms are considerably different, with cropping creating more pollution and soil erosion, while both systems incur unavoidable greenhouse gas emissions. In this case, the relevant consumer choice is likely to be between direct (Type 2) harm to one species of wildlife via harvesting or indirect (Type 3 and Type 4) harms to many species of wildlife through cropping.

Vexing harm conflicts can occur even when considering the best way to consume one type of food. Consider the trade-off between plastic waste (Type 3) and food waste (Type 4) for fresh produce (i.e., fruit and vegetables). Forgoing plastic packaging from produce may reduce entanglement risks for wildlife but comes with a reduction in shelf life and the resultant increase in food waste [278], with far-reaching impacts on food webs.

### 5.2. Once-Off and Ongoing Harms

Some harms were inflicted in the past (e.g., land clearing and environmentally persistent toxins such as organochlorines) while others are ongoing (e.g., painful but routine livestock husbandry practices such as mulesing). For example, food safety testing (Section 3.2.4) generally only needs to be performed once for each chemical. As such, harms may not apply through this process when already-registered chemicals are being used. With Type 4 harms such as climate change and eutrophication of coastal areas, mitigation may be impossible after the initial disturbance has occurred. This distinction may be important to the cumulative impact created by different types of harms. For example, land clearing may impose enormous harms to wild animals per square meter of land [21] but will (typically) happen only once. Tilling, on the other hand, as used for annual crops, may impose fewer harms per square meter of land but may happen every year for the immediate future [279]. These impacts are difficult to compare quantitatively, but it is important to recognize that they differ in their cumulative impacts, with once-off processes typically imposing less harm than ongoing or repeated processes.

### 5.3. Quantity of Food Produced

So far in our discussion, all foods are treated equally regarding their nutritive value and efficiency of production, when in reality, they are not equal. Some food types have a relatively high content of energy, protein, and important nutrients (e.g., eggs), while others do not (e.g., mushrooms). Minimizing harms while maintaining (or increasing) food production will be a key global challenge in the future [280]. For this reason, some analyses of food production systems have compared not only equivalent masses of produce (i.e., kilograms of food), but have looked at, for example, equivalent masses of useable protein (i.e., kilograms of protein) [84]. When relatively nutrient-poor foods such as mushrooms are examined on this basis, they cannot realistically be proposed as an alternative diet choice for some other foods. It would be exceedingly difficult (if not impossible) to sustain a group of hundreds, let alone billions, of humans solely on harvested mushrooms. Hence, if individual people are particularly concerned about animal harms, then there are food alternatives that they can utilize to some degree (e.g., wild mushrooms), but wholesale shifts towards these alternatives by large groups of people are unlikely and will not necessarily alleviate animal harm. For this reason, it may be more useful to compare common protein-rich foods because they could be seen more as realistic alternative diet choices. The efficiency of food production also determines not only the range of ways in which animals are harmed, but how many animals are harmed.

### 5.4. Numbers of Animals Harmed

Another crucial consideration when attempting any sort of quantitative comparison of animal welfare impacts is the number of animals affected [11]. When this consideration is applied to food production, a metric of number of animals harmed per unit of food can be conceptualized. This is not necessarily simple arithmetic due to the diversity in types of harms involved and no obvious way to weigh each of them. However, some interesting trends appear when animal size is factored in. For very large animals that produce very large amounts of food (e.g., whales), economies of scale become apparent. Conversely, for very small animals harmed during food production (e.g., rodents), a large number of animals may need to be harmed for a modest quantity of food to be produced. For example, one harvested large whale, such as a southern right whale (*Eubalaena australis*) may produce approximately 30 tons of food [281] for one sentient animal that is harmed. At the other end of the spectrum, estimates of rodent killing (via poisoning) required during house mouse (*Mus musculus*) plagues in Australian crop lands estimate that >500 mice are intentionally harmed per ton of useable wheat [84], or ~15,000 mice instead of 1 whale. This raises the question of whether harms to whales are morally equivalent to harms to mice.

### 5.5. Animal Sentience and Hierarchies of Intelligence

As we suggested earlier (Section 1), there is disagreement about whether all animals are equally morally important. However, this need not be understood as an arbitrary privileging of one species over another. Instead, according to one standard way of framing these issues, animals differ in their “capacity for welfare”—that is, how well or poorly their lives can go for them, which can also be understood as a measure of the richness and variety of the experiences they can have [11]. So, it might be thought that whales have a much greater capacity for welfare than mice, perhaps to the point that one whale is “worth” many mice. This, however, is just a way of saying that, given their differences in capacity for welfare, whales have far more to lose in death than mice do.

At some point along this hierarchy of animal sentience, it may be sufficiently unlikely that certain animals have any capacity for welfare that it is appropriate not to factor their (possible) welfare in moral deliberations. If so, then there is a point at which the numbers become moot. So, while it might be important to consider trade-offs between, say, whales and mice, it may not be important to consider trade-offs between whales and insects. Not incidentally, this view provides support for the use of insect meal as a source of protein despite the vast numbers of insects that need to be slaughtered to produce significant quantities of protein but can be tempered depending on the food source(s) provided to the insects.

### 5.6. Area Affected

When Type 3 and 4 harms are considered, and attempts are made to quantify unintentional harms to wildlife, much hinges on the amount of land (or water) area occupied by different production systems. Land area used by different production systems vary widely, with some industries (e.g., edible insects) occupying negligible land area, and others (e.g., rangeland pastoralism) occupying enormous areas. For example, in Australia, rangeland pastoralism accounts for 71% of all agricultural land use, or approximately 50% of Australia’s land mass [282]. Type 1 and 2 harms are usually constrained to relatively few species, small areas, and small numbers of animals, whereas Type 3 and 4 harms (e.g., climate change and desertification) are global in scale and affect countless numbers of individuals, numerous species and whole ecosystems.

### 5.7. Which Animals and Which Harms to Prioritize

Given that all food production systems cause harm to animals, the logical question for a concerned consumer is which harms should be considered more important than others. Are domestic animals more important than wild animals? Are mammals more important than birds? Are endangered species more important than common species? Should intentional harms be avoided more strongly than unintentional harms? Is it better to harm a lot of animals only a little, or to harm only a few animals a lot? Should we prioritize the few animals likely to experience the most severe harms or the greatest number of animals likely to experience less severe harms? There are no scientific approaches that can answer whether it is better to kill one whale for 30 tons of meat versus killing 15,000 mice for 30 tons of grain. Instead, we must look to ethics to un-pack the questions being asked here.

Some consumers might think that, due to differences in cognitive sophistication, less intense harms to orangutans as a result of palm oil production are still more weighty, morally speaking, than more intense harms to rodents in crop production. Others, however, might object to privileging species based on the degree of cognitive sophistication, insisting that only the capacity for pleasure and pain should count. These examples show that the first step is simply to make the catalogue of harms available for discussion, as we did here. Depending on the particular dietary options under consideration, further research will be required to determine how those choices should be made.

Assuming an egalitarian position, whereby all sentient animals assume equivalent moral status, intensive animal industries that require extensive modifications to land use (e.g., dairy) are associated with the broadest range of animal harms, whereas harvesting wild plants, mushrooms and seaweed are associated with the narrowest range of harms. Some of the outputs of our analysis are less intuitive. For instance, when choosing between wildlife that has been professionally harvested or eating plants from intensive crop production (Section 5.1), our breadth analysis indicates that game meat consumption is associated with fewer harms than some plant-based diets (Table 1).

## 6. Decision Making for Consumers

Given the above analysis, what should consumers do? As we suggested at the outset, the answer is not as simple as choosing the food type with the least breadth of harms. That is only part of the animal welfare picture and animal welfare is only one of many ethical concerns relevant to food choices [276]. Ultimately, any decision made on the part of the consumer will depend on what they personally prioritize.

For our purposes, a consumer ethic is a theory about what consumers ought to do, all things considered. It is not simply a theory about what consumers ought to do given one particular objective, such as minimizing animal harms or carbon footprints, or one of the many other morally significant aims [283]. As a result, a consumer ethic needs to tell consumers how to balance these diverse goals. And to do that, it needs to say something about their relative significances. In other words, it needs to provide some sort of ranking so that consumers know what to prioritize when they face conflict and trade-offs between legitimate moral aims, as they so often do.

To provide that ranking, a consumer ethic depends on more general theorizing about the relative moral significance of different kinds of harms, the relative moral significance of human and nonhuman animals, the moral significance of the environment, and much else besides. For instance, we might be concerned about how harm is distributed — that is, the overall pattern of harm. Maybe there are special reasons to be worried, morally speaking, about highly concentrated harms in the way that focus might be applied to overlapping kinds of oppression, or harms to groups that have suffered various injustices in the past (in the way concerns are amplified for harms to Indigenous human populations). So, for instance, while it might be the case that mice suffer a great deal when poisoned, and more than orangutans do as a result of habitat loss from palm oil production, the fact that orangutans are critically endangered and mice are not may mean that harms to orangutans deserve special attention — not because species losses are bad in itself, but because harms are being concentrated on a species that has already lost so much.

There are also epistemic hurdles that consumers face. Modern consumers are confronted with an enormous amount of information and conflicting opinions about what they ought to purchase and eat, and even regulated information is often difficult for consumers to interpret. Consider, for instance, the gap between regulatory standards and public perceptions when it comes to labels like “organic”, “free range” [284] or “eco-labels” [285]. This problem is particularly acute when it comes to animal welfare, as many consumers anthropomorphize animals in ways that make consumers misjudge the trade-offs in various production systems (e.g., caged versus cage-free systems for chickens) [286]. 

On top of this, there are many people who do not have the luxury of choosing how their food is produced. And for most of those who do have that luxury, it only comes in degrees. Consumers at different points of the income scale have more and fewer opportunities to adjust their purchasing in response to moral considerations, which means that they face different sets of options from which they must make their selections. Each set leaves consumers with a distinct set of trade-offs that need to be adjudicated which, given the epistemic challenges just mentioned, can be highly cognitively taxing.

A usable consumer ethic needs to provide people with some sense of what they ought to do when the relevant moral and empirical facts are difficult to assess. In other words, a good consumer ethic will make it possible to generate “rules of thumb” that, while delivering imperfect advice about how to shop and eat, provide advice that is good enough for navigating a fraught food system. Again, we take no position on any of the substantive questions here and we are not advocating for any particular consumer ethic, general ethical theory, or other moral position. Instead, we simply want to stress the importance of moral theorizing for generating a consumer ethic while noting that all food production systems cause unavoidable harm to some animals. It is indeed important to catalogue the breadth of harms to nonhuman animals, but that is only the first step.

## 7. Where to from Here?

We recognize that there is enormous diversity in food production practices and that the conclusions we draw will not be universal. For example, many of the harms we identified are only relevant to certain geographic locations. Increasing use of recently developed techniques such as zero-tillage cropping [287] or lead-free bullets for wildlife harvesting [181] may reduce or eliminate harms that currently occur within conventional practices. We also do not intend for our conclusions to remain fixed. New food technologies such as cellular agriculture are likely to require a re-evaluation of the comparative benefits of different systems as new products become more widely available [266]. However, given the inescapable connection between sentient animals and the ecosystems from which we obtain our food, we do not expect to see a “harm-free” food option emerge in the foreseeable future. When applied to food choices, deontological concepts such as “do no harm” [288] would seem to be impossible once Type 3 and Type 4 harms are considered [289].

We also acknowledge that consumer choices are only one part of a complex picture determining how food is produced, and what harms are imposed on animals. In addition to consumers, the decisions made by food producers, processors, retailers and policymakers have strong influences [1]. We further acknowledge that this paper focuses exclusively on the breadth of harms associated with different foods and ignores intensity, but we expect that our defined breadth of harms can be subsequently populated with the estimates of intensity of each harm in the future. Our goal here was not to make recommendations about how to eat, but rather to indicate how much research remains to be done before we can determine which ways to eat or harm the fewest animals. Our focus is also not exclusively on the choice between eating products that are derived from intensive animal agriculture and eating in less conventional ways. Instead, we suggest that concerned consumers carefully examine their own system of ethics in order to answer the question of what they ought to eat for themselves. Much research remains to be done on the indirect impacts of food production on animals but the trend is clear: human activities are affecting all animal life in more ways than we currently understand, and there are no present options for producing foods that are “harm-free”. Some difficult decisions are required for anyone concerned with minimizing their impacts on animals. At a fundamental level, the best we may be able to do is choose a balance of products that do not intentionally harm animals (but do anyway) and those that do intentionally harm animals (but create a lesser breadth of harms).

Our analysis further highlights the close connection between animal welfare and biodiversity concerns when animal welfare is considered broadly to include Type 4 harms and wild animals are given due consideration. A philosophical conclusion may be that we should replace our current approach to ethics, where animal ethics and environmental ethics are often discussed in isolation from each other, and develop a coherent, over-arching ethic for animals that includes the harms we cause to other species, whether intentionally or unintentionally, and whether directly or indirectly [290]. These two fields will need to intersect more closely and use a common language in the future to ensure that humans can live and eat sustainably while attempting to minimize our impacts on individual animals [290]. The tenets of “One Welfare” [12], recognizing the connection between human, animal and environmental welfare, may be a step in this direction.

## 8. Conclusions

We show that all food production systems harm animals and that most harms imposed by food production have unintentional or indirect effects on wildlife. Some food production systems incur very few animal harms (harvesting of wild plants, seaweeds, and mushrooms), while animal agriculture systems that require the feeding of crops could be argued to impose the greatest range of harms to the greatest number of animals. We encourage affluent consumers to carefully consider their own ethical stance toward different animals and use consideration of harms as a guide for selecting food rather than relying on activism, marketing, or advocacy. With world food production systems facing a challenge to increase production by approximately 70% during the next 30 years [291], much will change. If nothing else, we hope that this paper will create basic recognition of these broad issues so that they can be taken into account in moral decision-making. We acknowledge that animal welfare is one of many factors to consider for consumers selecting food types. We hope that our analysis may also be informative to food producers, processors, retailers and policymakers concerned with animal welfare. This perspective may, at the least, provide “food for thought” for contemporary consumers who place a high priority on animal welfare.

## Figures and Tables

**Table 1:** Harms associated with food production systems. Shading indicates frequency of harms: white = none, light grey = inconsistent, and dark grey = consistent.

Food Production System	Secondary System Required *	Harms																				
		Type 1			Type 2				Type 3					Type 4								
		H	LT	WA	LS	WH	WDM	FST	LC	TH	E	I	TE	P	GG	IS	PD	ID	S	SE	FW	DNR
Plant, mushroom, seaweed harvest	NA																					
Apiary	NA																					
Terrestrial wildlife harvest	NA																					
Marine wildlife harvest	NA																					
Extensive egg production	NA																					
Rangeland pastoralism	NA																					
Dryland cropping	NA																					
Horticulture	NA																					
Irrigated cropping	NA																					
Edible insects	Cropping, fishing, aquaculture																					
Cellular agriculture	Cropping, livestock																					
Aquaculture/ mariculture	Fishing																					
Intensive egg production	Cropping, edible insects																					
Extensive livestock	NA																					
Intensive livestock	Cropping																					
Dairy	Cropping																					

H = Husbandry, LT = Livestock Transport, WA = Working Animals, LS = Livestock Slaughter, WH = Wildlife Harvesting, WDM = Wildlife Damage Management, FST = Food Safety Testing, LC = Land Clearing, TH = Tillage and Harvesting, E = Entanglement, I = Irrigation, TE = Transport Effects, P = Pollution, GG = Greenhouse Gasses, IS = Invasive Species, PD = Predation of Domestic Animals, ID = Infectious Diseases, S = Salinity, SE = Soil Erosion, FW = Food Waste, DNR = Depletion of Natural Resources. * All harms associated with the secondary system required will also be incurred (i.e., for these systems, harms are additive). NA = Not applicable.
